# Supplementary material for: Simultaneous Detection of Porcine Respiratory Coronavirus, Porcine Reproductive and Respiratory Syndrome Virus, Swine Influenza Virus, and Pseudorabies Virus via Quadruplex One-Step RT-qPCR
Source: Pathogens. 2024 Apr 19;13(4):341. doi: 10.3390/pathogens13040341 (PMC11054806; doi:10.3390/pathogens13040341)
Supplement: Supplementary file 1 [file pathogens-13-00341-s001.zip › pathogens-2896401-supplementary.pdf]

Supplementary Figure S1

A. PRCoV

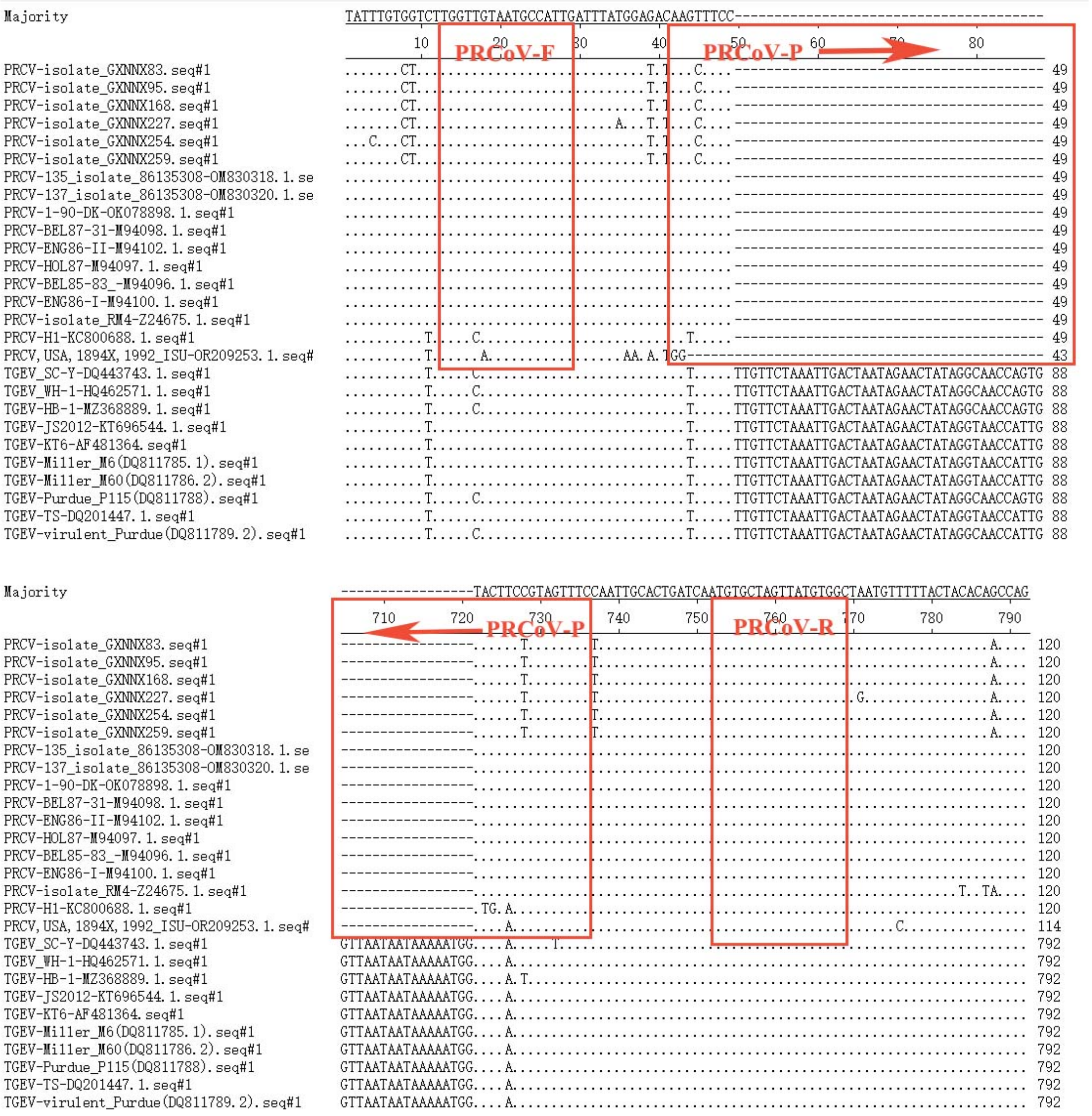

B. PRRSV

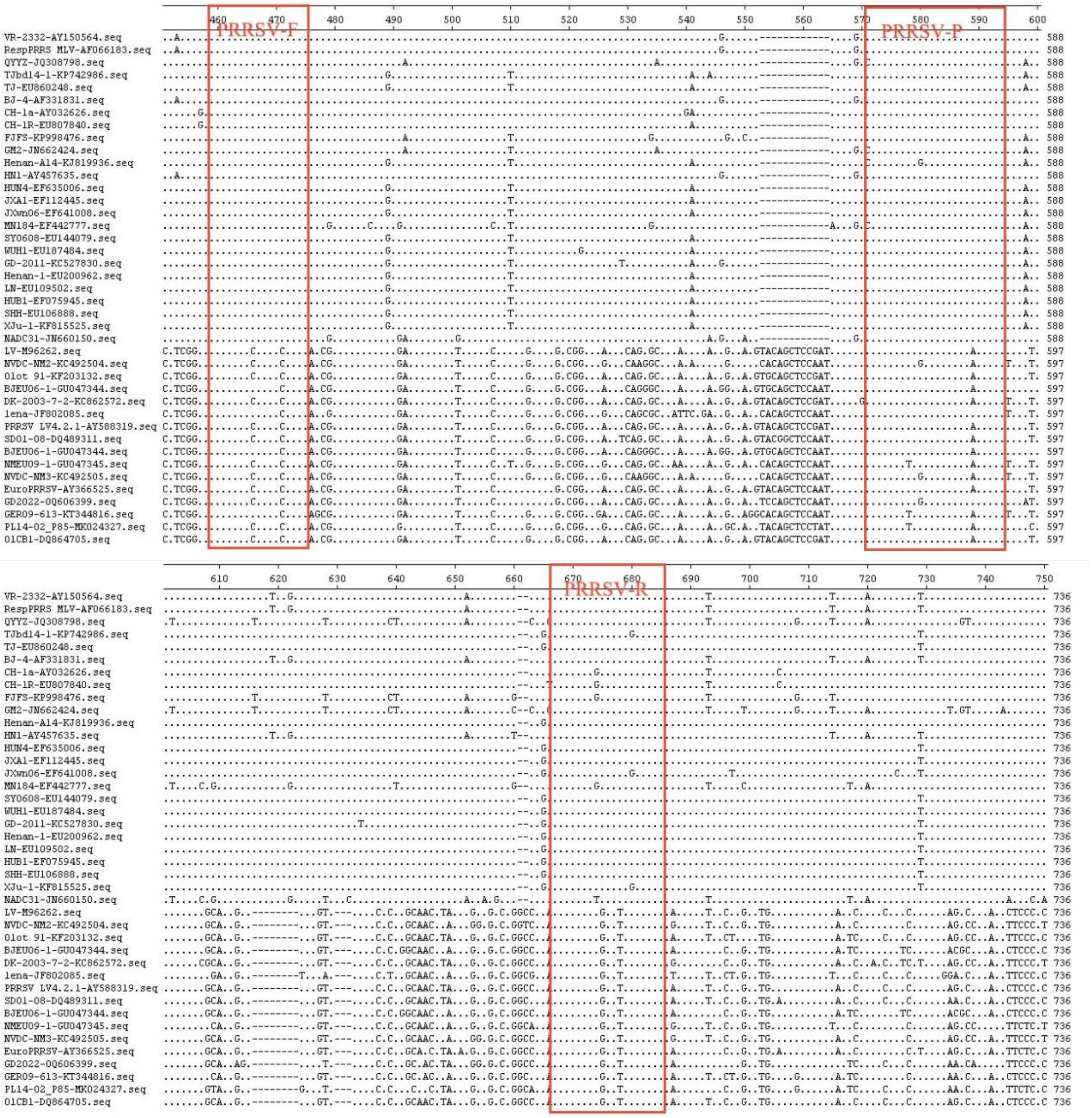

C. SIV

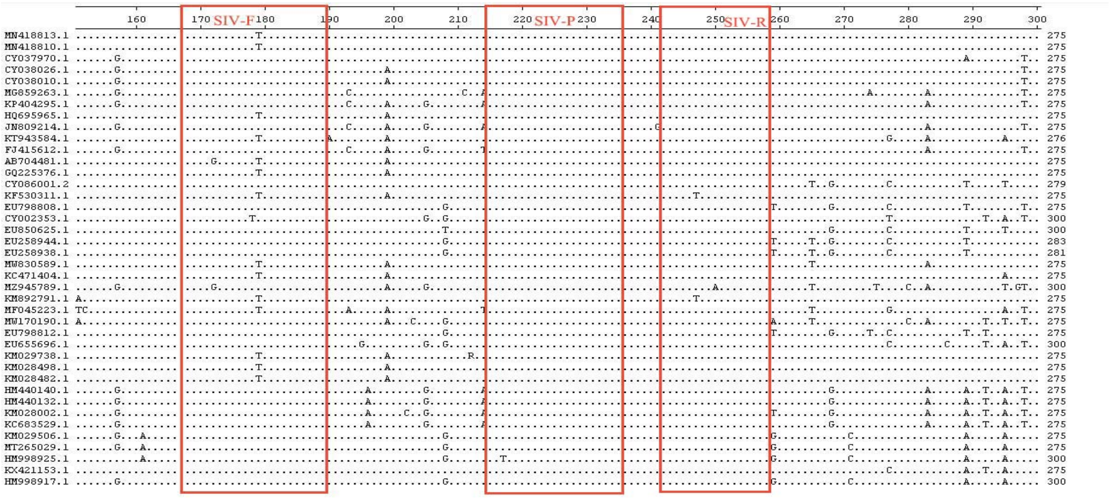

D. PRV

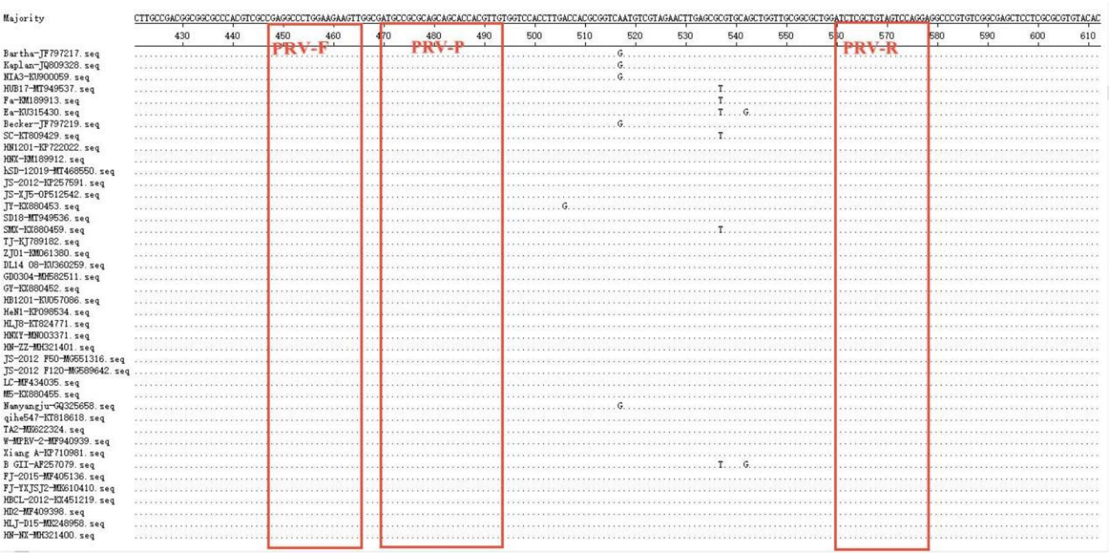

Figure S1. Location of the specific primers and probes. The primers and TaqMan probes were designed basing on the conserved regions of for PRCoV S gene (A), PRRSV N gene (B), SIV M gene (C), and PRV gB gene (D), respectively.
